# *N*-[(2-Hydr­oxy-1-naphthyl)(3-nitro­phenyl)meth­yl]acetamide

**DOI:** 10.1107/S1600536809007442

**Published:** 2009-03-06

**Authors:** M. NizamMohideen, A. SubbiahPandi, N. Panneer Selvam, P. T. Perumal

**Affiliations:** a Department of Physics, The New College (Autonomous), Chennai 600 014, India; b Department of Physics, Presidency College (Autonomous), Chennai 600 005, India; c Organic Chemistry Division, Central Leather Research Institute, Chennai 600 020, India

## Abstract

The title compound, C_19_H_16_N_2_O_4_, is of inter­est as a precursor to biologically active substituted quinolines and related compounds. The dihedral angle between the naphthalene ring system and the benzene ring is 81.9 (1)°. The crystal structure is stabilized by N—H⋯O inter­molecular hydrogen bonds, linking the mol­ecules into pairs around a center of symmetry. The crystal structure is further stabilized by inter­molecular O—H⋯O hydrogen bonds, which link the mol­ecules into chains running along *a* axis. An intra­molecular C—H⋯O short contact is also present.

## Related literature

For *N*-(substituted phen­yl)acetamides as precursors for the synthesis of hetrocyclic compounds, see: Wen *et al.* (2005 ▶, 2006 ▶). For multicomponent reactions, see: Devi & Bhuyan (2004 ▶); Domling & Ugi (2000 ▶). For the properties and potential applications of amide-type compounds and their metal ion complexes, see: Saravanakumar *et al.* (2005 ▶); Yin *et al.* (2004 ▶). For related structures, see: Mosslemin *et al.* (2007 ▶); Zia-ur-Rehman *et al.* (2008 ▶). For bond-length data, see: Allen *et al.* (1987 ▶); Liu & Li (2004 ▶). For hydrogen-bond motifs, see: Bernstein *et al.* (1995 ▶).
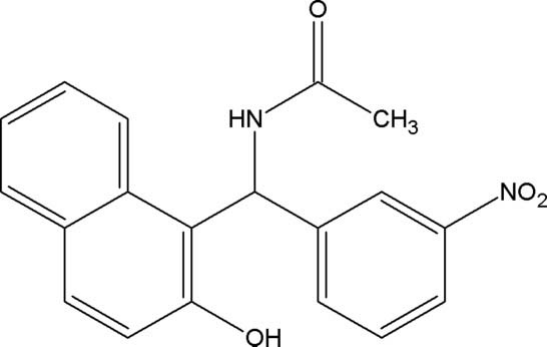


## Experimental

### 

#### Crystal data

C_19_H_16_N_2_O_4_
*M* *_r_* = 336.34
Triclinic, 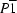

*a* = 7.5261 (4) Å
*b* = 8.8635 (5) Å
*c* = 13.3008 (7) Å
α = 74.720 (3)°
β = 73.754 (3)°
γ = 82.600 (3)°
*V* = 820.27 (14) Å^3^
*Z* = 2
Mo *K*α radiation
μ = 0.10 mm^−1^
*T* = 293 K
0.4 × 0.2 × 0.1 mm

#### Data collection

Bruker Kappa APEXII CCD diffractometer
Absorption correction: multi-scan (*SADABS*; Bruker, 2004 ▶) *T*_min_ = 0.974, *T*_max_ = 0.990
9406 measured reflections
3864 independent reflections
2227 reflections with *I* > 2σ(*I*)
*R*_int_ = 0.026

#### Refinement

*R*[*F* ^2^ > 2σ(*F* ^2^)] = 0.046
*wR*(*F* ^2^) = 0.144
*S* = 0.99
3864 reflections
227 parameters
H-atom parameters constrained
Δρ_max_ = 0.21 e Å^−3^
Δρ_min_ = −0.17 e Å^−3^

### 

Data collection: *APEX2* (Bruker, 2004 ▶); cell refinement: *APEX2* and *SAINT* (Bruker, 2004 ▶); data reduction: *SAINT* and *XPREP* (Bruker, 2004 ▶); program(s) used to solve structure: *SHELXS97* (Sheldrick, 2008 ▶); program(s) used to refine structure: *SHELXL97* (Sheldrick, 2008 ▶); molecular graphics: *ORTEP-3 for Windows* (Farrugia, 1997 ▶); software used to prepare material for publication: *SHELXL97* and *PLATON* (Spek, 2009)’.

## Supplementary Material

Crystal structure: contains datablocks global, I. DOI: 10.1107/S1600536809007442/jh2076sup1.cif

Structure factors: contains datablocks I. DOI: 10.1107/S1600536809007442/jh2076Isup2.hkl

Additional supplementary materials:  crystallographic information; 3D view; checkCIF report

## Figures and Tables

**Table 1:** Hydrogen-bond geometry (Å, °)

*D*—H⋯*A*	*D*—H	H⋯*A*	*D*⋯*A*	*D*—H⋯*A*
O1—H1⋯O4^i^	0.82	1.87	2.646 (2)	158
N1—H1*A*⋯O2^ii^	0.86	2.35	3.167 (2)	160
C11—H11⋯O4	0.98	2.28	2.739 (2)	107

Symmetry codes: (i) 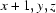; (ii) 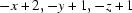.

**Fig. 1.**
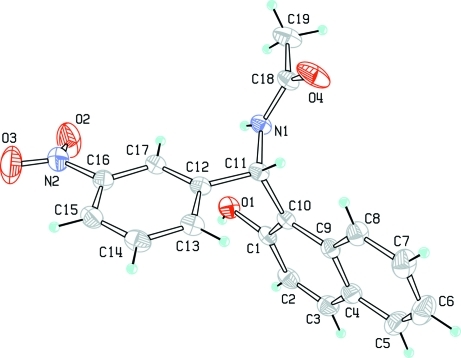
The molecular configuration and atom-numbering scheme for (I). Displacement ellipsoids are drawn at the 30% probability level.

**Fig. 2.**
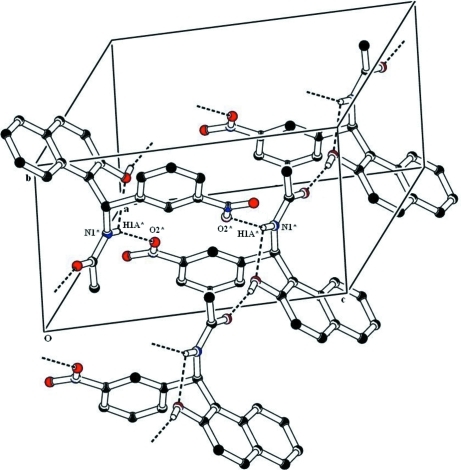
Part of the crystal structure of (I), showing the R22(16) rings. For the sake of clarity, H atoms not participating in the hydrogen bonding have been omitted. Hydrogen bonding is shown as dashed lines. [Symmetry codes: (*) -x + 2, -y + 1, -z + 1]

## supplementary crystallographic information

### Comment

N-(substituted phenyl)acetamides are well known for their importance as intermediates in organic synthesis. They are used as precursors for the synthesis of many hetrocyclic compounds (Wen *et al.*, 2005, 2006). Multi-component reactions (MCRs) have attracted considerable attention in terms of the saving of both energy and raw materials (Devi & Bhuyan, 2004). They have merits over multi-step reactions in several aspects, including the simplicity of a one-pot procedure, possible structural variations and in building up complex molecules (Domling & Ugi, 2000). Much attention has been focused on amide-type compounds and their metal ion complexes for their properties and potential applications including molecular recognition, ion electrodes, photochemistry and topological structures in ion extraction, biochemistry, catalysis and magnetism (Saravanakumar *et al.*, 2005; Yin *et al.*, 2004). The amide linkage [–NHC(O)-] is known to be strong enough to form and maintain protein architectures and has been utilized to create various molecular devices for a spectrum of purposes in organic chemistry. In order to obtain fundamental information about this phenomenon, an X-ray crystal structure analysis of (I) was undertaken.

The conformation of (I), together with the atom-numbering scheme, is shown in Fig. 1. In the structure, all bond lengths and angles are within normal ranges (Allen *et al.*, 1987), and comparable with those in previously reported structure (Mosslemin *et al.*, 2007). The bond distance of C18=O4 is 1.222 (2) Å, which is typical for double bonds (Liu & Li., 2004). The nitro group is slighty twisted out of the plane of the benzene ring, as indicated by O2—N2—C16—C15 and O3—N2—C16—C15 torsion angles of -162.9 (2) and 14.4 (3)°, respectively, and comparable with those in previously reported structure (Zia-ur-Rehman *et al.*, 2008).

The naphthalene ring is planar, the maximum deviation from the least squares plane being -0.027 (1) Å for atom C7. Atom O1 deviating by 0.05 (1) Å from the least squares plane of the naphthalene ring. The dihedral angle between the naphthalene and benzene ring is 81.9 (1)°. The dihedral angle between the fused rings is 1.1 (1)°.

The crystal structure is stabilized by N—H···O intermolecular hydrogen bonds (Table 1, Fig 2.) that generate centrosymmetric hydrogenbonded dimers with a cyclic *R*^2^_2_(16) ring system (Bernstein, *et al.*, 1995). The crystal structure is further stabilized by intermolecular O—H···O hydrogen bonds link the molecules into chains running along *a* axis.

### Experimental

A mixture of 3-nitrobenzaldehyde (10 mmol), β-naphthol (10 mmol) and iodine (0.4 mmol, 4 mol%) were mixed in acetonitrile (5 ml). To that suspension acetyl chloride (2.8 mmol, 0.2 ml) was added and the reaction mixture was stirred at room temperature for 3 h. After the completion of the reaction (as monitored by TLC), saturated sodium thiosulfate solution (5 ml) was added. The precipitated solid was filtered and dried. The dried sample was washed with diethyl ether (2 *x* 10 ml) and again dried. Single crystals of the title compound suitable for X-ray diffraction were obtained by slow evaporation of a solution in Ethanol.

### Refinement

All H atoms were positioned geometrically, with N—H = 0.86 and C—H = 0.93, 0.98 and 0.96 Å aromatic, methylene and methyl H, respectively, and constrained to ride on their parent atoms, with *U*_iso_(H) = *xU*_eq_(C, N), where *x* = 1.5 for methyl H, and *x* = 1.2 for all other H atoms.

### Crystal data

**Table:**

C_19_H_16_N_2_O_4_	*Z* = 2
*M**_r_* = 336.34	*F*(000) = 352
Triclinic, *P*1	*D*_x_ = 1.362 Mg m^−^^3^
Hall symbol: -P 1	Mo *K*α radiation, λ = 0.71073 Å
*a* = 7.5261 (4) Å	Cell parameters from 3864 reflections
*b* = 8.8635 (5) Å	θ = 2.5–25°
*c* = 13.3008 (7) Å	µ = 0.10 mm^−^^1^
α = 74.720 (3)°	*T* = 293 K
β = 73.754 (3)°	Needle, colourless
γ = 82.600 (3)°	0.4 × 0.2 × 0.1 mm
*V* = 820.27 (14) Å^3^	

### Data collection

**Table:**

Bruker Kappa APEXII CCD diffractometer	3864 independent reflections
Radiation source: fine-focus sealed tube	2227 reflections with *I* > 2σ(*I*)
graphite	*R*_int_ = 0.026
ω and φ scan	θ_max_ = 28.4°, θ_min_ = 2.6°
Absorption correction: multi-scan (*SADABS*; Bruker, 2004)	*h* = −9→10
*T*_min_ = 0.974, *T*_max_ = 0.990	*k* = −11→11
9406 measured reflections	*l* = −17→17

### Refinement

**Table:**

Refinement on *F*^2^	Primary atom site location: structure-invariant direct methods
Least-squares matrix: full	Secondary atom site location: difference Fourier map
*R*[*F*^2^ > 2σ(*F*^2^)] = 0.046	Hydrogen site location: inferred from neighbouring sites
*wR*(*F*^2^) = 0.144	H-atom parameters constrained
*S* = 0.99	*w* = 1/[σ^2^(*F*_o_^2^) + (0.0612*P*)^2^ + 0.220*P*] where *P* = (*F*_o_^2^ + 2*F*_c_^2^)/3
3864 reflections	(Δ/σ)_max_ = 0.009
227 parameters	Δρ_max_ = 0.21 e Å^−^^3^
0 restraints	Δρ_min_ = −0.17 e Å^−^^3^

### Special details

**Table:**

Geometry. All e.s.d.'s (except the e.s.d. in the dihedral angle between two l.s. planes) are estimated using the full covariance matrix. The cell e.s.d.'s are taken into account individually in the estimation of e.s.d.'s in distances, angles and torsion angles; correlations between e.s.d.'s in cell parameters are only used when they are defined by crystal symmetry. An approximate (isotropic) treatment of cell e.s.d.'s is used for estimating e.s.d.'s involving l.s. planes.
Refinement. Refinement of *F*^2^ against ALL reflections. The weighted *R*-factor *wR* and goodness of fit *S* are based on *F*^2^, conventional *R*-factors *R* are based on *F*, with *F* set to zero for negative *F*^2^. The threshold expression of *F*^2^ > σ(*F*^2^) is used only for calculating *R*-factors(gt) *etc*. and is not relevant to the choice of reflections for refinement. *R*-factors based on *F*^2^ are statistically about twice as large as those based on *F*, and *R*- factors based on ALL data will be even larger.

### Fractional atomic coordinates and isotropic or equivalent isotropic displacement parameters (Å^2^)

**Table:**

	*x*	*y*	*z*	*U*_iso_*/*U*_eq_	
O1	1.11375 (16)	0.78003 (16)	0.26550 (11)	0.0490 (4)	
H1	1.2259	0.7587	0.2525	0.074*	
O2	0.9758 (4)	0.5649 (2)	0.62604 (17)	0.1025 (7)	
O3	0.8781 (4)	0.6954 (3)	0.74538 (16)	0.1150 (8)	
O4	0.46636 (19)	0.7392 (2)	0.26984 (17)	0.0801 (6)	
N1	0.74868 (19)	0.71417 (18)	0.29573 (12)	0.0401 (4)	
H1A	0.8443	0.6528	0.3045	0.048*	
N2	0.8965 (3)	0.6794 (3)	0.65559 (16)	0.0667 (5)	
C1	1.0777 (2)	0.9135 (2)	0.19395 (14)	0.0385 (4)	
C2	1.2195 (2)	0.9945 (3)	0.11175 (16)	0.0502 (5)	
H2	1.3425	0.9575	0.1056	0.060*	
C3	1.1769 (3)	1.1262 (3)	0.04174 (17)	0.0536 (5)	
H3	1.2720	1.1793	−0.0117	0.064*	
C4	0.9922 (3)	1.1848 (2)	0.04788 (15)	0.0473 (5)	
C5	0.9457 (4)	1.3208 (3)	−0.02594 (17)	0.0632 (6)	
H5	1.0402	1.3758	−0.0786	0.076*	
C6	0.7679 (4)	1.3729 (3)	−0.0220 (2)	0.0732 (7)	
H6	0.7408	1.4619	−0.0721	0.088*	
C7	0.6253 (4)	1.2929 (3)	0.05736 (19)	0.0634 (6)	
H7	0.5029	1.3285	0.0595	0.076*	
C8	0.6626 (3)	1.1628 (2)	0.13215 (17)	0.0495 (5)	
H8	0.5649	1.1126	0.1852	0.059*	
C9	0.8474 (2)	1.1027 (2)	0.13058 (14)	0.0384 (4)	
C10	0.8943 (2)	0.9665 (2)	0.20492 (14)	0.0353 (4)	
C11	0.7483 (2)	0.8774 (2)	0.29803 (14)	0.0356 (4)	
H11	0.6276	0.9264	0.2889	0.043*	
C12	0.7637 (2)	0.8970 (2)	0.40573 (14)	0.0366 (4)	
C13	0.7214 (2)	1.0446 (2)	0.42690 (16)	0.0448 (5)	
H13	0.6865	1.1272	0.3751	0.054*	
C14	0.7299 (3)	1.0718 (3)	0.52306 (17)	0.0508 (5)	
H14	0.6998	1.1715	0.5356	0.061*	
C15	0.7827 (3)	0.9515 (3)	0.60021 (16)	0.0500 (5)	
H15	0.7880	0.9677	0.6656	0.060*	
C16	0.8275 (3)	0.8069 (2)	0.57761 (15)	0.0452 (5)	
C17	0.8192 (2)	0.7764 (2)	0.48202 (14)	0.0411 (4)	
H17	0.8504	0.6766	0.4697	0.049*	
C18	0.6061 (2)	0.6567 (2)	0.28056 (16)	0.0457 (5)	
C19	0.6235 (3)	0.4876 (3)	0.2787 (2)	0.0719 (7)	
H19A	0.5450	0.4293	0.3438	0.108*	
H19B	0.7499	0.4483	0.2734	0.108*	
H19C	0.5866	0.4764	0.2176	0.108*	

### Atomic displacement parameters (Å^2^)

**Table:**

	*U*^11^	*U*^22^	*U*^33^	*U*^12^	*U*^13^	*U*^23^
O1	0.0284 (6)	0.0571 (9)	0.0628 (9)	0.0062 (6)	−0.0192 (6)	−0.0126 (7)
O2	0.163 (2)	0.0682 (13)	0.0971 (15)	0.0310 (13)	−0.0825 (15)	−0.0215 (12)
O3	0.189 (3)	0.1062 (17)	0.0557 (12)	0.0012 (16)	−0.0525 (14)	−0.0122 (11)
O4	0.0403 (8)	0.0771 (12)	0.1474 (17)	0.0087 (8)	−0.0497 (10)	−0.0471 (12)
N1	0.0309 (7)	0.0380 (9)	0.0574 (9)	0.0045 (6)	−0.0211 (7)	−0.0146 (7)
N2	0.0861 (14)	0.0639 (14)	0.0559 (12)	−0.0130 (11)	−0.0321 (10)	−0.0056 (11)
C1	0.0318 (8)	0.0457 (11)	0.0440 (10)	0.0000 (8)	−0.0138 (7)	−0.0177 (9)
C2	0.0313 (9)	0.0701 (14)	0.0556 (12)	−0.0066 (9)	−0.0079 (8)	−0.0277 (11)
C3	0.0516 (12)	0.0623 (14)	0.0462 (11)	−0.0172 (10)	−0.0023 (9)	−0.0161 (11)
C4	0.0581 (12)	0.0454 (12)	0.0416 (10)	−0.0068 (9)	−0.0100 (9)	−0.0170 (9)
C5	0.0878 (18)	0.0488 (13)	0.0467 (12)	−0.0081 (12)	−0.0087 (12)	−0.0080 (11)
C6	0.104 (2)	0.0478 (14)	0.0603 (15)	0.0171 (14)	−0.0250 (14)	−0.0070 (12)
C7	0.0736 (15)	0.0513 (13)	0.0669 (15)	0.0205 (12)	−0.0295 (12)	−0.0161 (12)
C8	0.0502 (11)	0.0455 (12)	0.0554 (12)	0.0086 (9)	−0.0204 (9)	−0.0145 (10)
C9	0.0424 (9)	0.0368 (10)	0.0410 (10)	0.0010 (8)	−0.0135 (8)	−0.0162 (8)
C10	0.0314 (8)	0.0386 (10)	0.0418 (10)	−0.0003 (7)	−0.0128 (7)	−0.0165 (8)
C11	0.0268 (8)	0.0343 (10)	0.0496 (10)	0.0039 (7)	−0.0154 (7)	−0.0131 (8)
C12	0.0238 (7)	0.0404 (10)	0.0462 (10)	−0.0016 (7)	−0.0079 (7)	−0.0126 (8)
C13	0.0374 (9)	0.0424 (11)	0.0554 (12)	0.0021 (8)	−0.0107 (8)	−0.0162 (9)
C14	0.0437 (10)	0.0500 (12)	0.0622 (13)	−0.0030 (9)	−0.0055 (9)	−0.0280 (11)
C15	0.0436 (10)	0.0636 (14)	0.0466 (11)	−0.0122 (10)	−0.0046 (9)	−0.0229 (11)
C16	0.0413 (10)	0.0504 (12)	0.0440 (11)	−0.0097 (9)	−0.0106 (8)	−0.0084 (9)
C17	0.0389 (9)	0.0405 (11)	0.0468 (11)	−0.0037 (8)	−0.0121 (8)	−0.0133 (9)
C18	0.0350 (9)	0.0511 (12)	0.0568 (12)	−0.0047 (8)	−0.0161 (8)	−0.0175 (10)
C19	0.0677 (15)	0.0586 (15)	0.103 (2)	−0.0135 (12)	−0.0270 (14)	−0.0323 (15)

### Geometric parameters (Å, °)

**Table:**

O1—C1	1.359 (2)	C7—H7	0.9300
O1—H1	0.8200	C8—C9	1.420 (3)
O2—N2	1.209 (3)	C8—H8	0.9300
O3—N2	1.207 (3)	C9—C10	1.420 (2)
O4—C18	1.222 (2)	C10—C11	1.520 (2)
N1—C18	1.329 (2)	C11—C12	1.525 (2)
N1—C11	1.455 (2)	C11—H11	0.9800
N1—H1A	0.8600	C12—C17	1.378 (2)
N2—C16	1.468 (3)	C12—C13	1.389 (3)
C1—C10	1.380 (2)	C13—C14	1.382 (3)
C1—C2	1.407 (3)	C13—H13	0.9300
C2—C3	1.353 (3)	C14—C15	1.375 (3)
C2—H2	0.9300	C14—H14	0.9300
C3—C4	1.408 (3)	C15—C16	1.372 (3)
C3—H3	0.9300	C15—H15	0.9300
C4—C5	1.414 (3)	C16—C17	1.387 (3)
C4—C9	1.428 (3)	C17—H17	0.9300
C5—C6	1.349 (3)	C18—O4	1.222 (2)
C5—H5	0.9300	C18—C19	1.494 (3)
C6—C7	1.391 (4)	C19—H19A	0.9600
C6—H6	0.9300	C19—H19B	0.9600
C7—C8	1.366 (3)	C19—H19C	0.9600
			
C1—O1—H1	109.5	C9—C10—C11	121.98 (14)
C18—N1—C11	122.19 (15)	N1—C11—C10	112.60 (14)
C18—N1—H1A	118.9	N1—C11—C12	112.92 (14)
C11—N1—H1A	118.9	C10—C11—C12	111.04 (13)
O3—N2—O2	122.6 (2)	N1—C11—H11	106.6
O3—N2—C16	118.8 (2)	C10—C11—H11	106.6
O2—N2—C16	118.6 (2)	C12—C11—H11	106.6
O1—C1—C10	116.89 (16)	C17—C12—C13	118.70 (17)
O1—C1—C2	122.07 (15)	C17—C12—C11	123.28 (16)
C10—C1—C2	121.03 (17)	C13—C12—C11	118.02 (16)
C3—C2—C1	120.01 (18)	C14—C13—C12	121.61 (19)
C3—C2—H2	120.0	C14—C13—H13	119.2
C1—C2—H2	120.0	C12—C13—H13	119.2
C2—C3—C4	121.59 (19)	C15—C14—C13	120.02 (19)
C2—C3—H3	119.2	C15—C14—H14	120.0
C4—C3—H3	119.2	C13—C14—H14	120.0
C3—C4—C5	122.1 (2)	C16—C15—C14	117.86 (18)
C3—C4—C9	118.75 (18)	C16—C15—H15	121.1
C5—C4—C9	119.13 (19)	C14—C15—H15	121.1
C6—C5—C4	121.8 (2)	C15—C16—C17	123.22 (19)
C6—C5—H5	119.1	C15—C16—N2	118.79 (18)
C4—C5—H5	119.1	C17—C16—N2	117.91 (19)
C5—C6—C7	119.7 (2)	C12—C17—C16	118.56 (18)
C5—C6—H6	120.2	C12—C17—H17	120.7
C7—C6—H6	120.2	C16—C17—H17	120.7
C8—C7—C6	121.0 (2)	O4—C18—N1	120.94 (18)
C8—C7—H7	119.5	O4—C18—N1	120.94 (18)
C6—C7—H7	119.5	O4—C18—C19	121.76 (18)
C7—C8—C9	121.4 (2)	O4—C18—C19	121.76 (18)
C7—C8—H8	119.3	N1—C18—C19	117.29 (17)
C9—C8—H8	119.3	C18—C19—H19A	109.5
C10—C9—C8	123.85 (17)	C18—C19—H19B	109.5
C10—C9—C4	119.07 (16)	H19A—C19—H19B	109.5
C8—C9—C4	117.07 (17)	C18—C19—H19C	109.5
C1—C10—C9	119.52 (16)	H19A—C19—H19C	109.5
C1—C10—C11	118.50 (15)	H19B—C19—H19C	109.5
			
O1—C1—C2—C3	179.40 (18)	C1—C10—C11—N1	−58.74 (19)
C10—C1—C2—C3	−0.2 (3)	C9—C10—C11—N1	122.18 (17)
C1—C2—C3—C4	−0.7 (3)	C1—C10—C11—C12	69.00 (19)
C2—C3—C4—C5	−178.9 (2)	C9—C10—C11—C12	−110.08 (17)
C2—C3—C4—C9	0.3 (3)	N1—C11—C12—C17	15.6 (2)
C3—C4—C5—C6	177.5 (2)	C10—C11—C12—C17	−111.95 (18)
C9—C4—C5—C6	−1.7 (3)	N1—C11—C12—C13	−165.13 (14)
C4—C5—C6—C7	0.9 (4)	C10—C11—C12—C13	67.30 (18)
C5—C6—C7—C8	0.6 (4)	C17—C12—C13—C14	−1.4 (3)
C6—C7—C8—C9	−1.2 (3)	C11—C12—C13—C14	179.28 (16)
C7—C8—C9—C10	−178.80 (19)	C12—C13—C14—C15	0.6 (3)
C7—C8—C9—C4	0.4 (3)	C13—C14—C15—C16	0.6 (3)
C3—C4—C9—C10	1.0 (3)	C14—C15—C16—C17	−1.0 (3)
C5—C4—C9—C10	−179.76 (18)	C14—C15—C16—N2	175.85 (18)
C3—C4—C9—C8	−178.19 (18)	O3—N2—C16—C15	14.4 (3)
C5—C4—C9—C8	1.0 (3)	O2—N2—C16—C15	−162.9 (2)
O1—C1—C10—C9	−178.11 (15)	O3—N2—C16—C17	−168.6 (2)
C2—C1—C10—C9	1.5 (3)	O2—N2—C16—C17	14.1 (3)
O1—C1—C10—C11	2.8 (2)	C13—C12—C17—C16	1.1 (2)
C2—C1—C10—C11	−177.60 (16)	C11—C12—C17—C16	−179.69 (16)
C8—C9—C10—C1	177.26 (17)	C15—C16—C17—C12	0.1 (3)
C4—C9—C10—C1	−1.9 (2)	N2—C16—C17—C12	−176.74 (16)
C8—C9—C10—C11	−3.7 (3)	C11—N1—C18—O4	−1.4 (3)
C4—C9—C10—C11	177.17 (16)	C11—N1—C18—O4	−1.4 (3)
C18—N1—C11—C10	−114.89 (18)	C11—N1—C18—C19	179.39 (19)
C18—N1—C11—C12	118.37 (18)		

### Hydrogen-bond geometry (Å, °)

**Table:**

*D*—H···*A*	*D*—H	H···*A*	*D*···*A*	*D*—H···*A*
O1—H1···O4^i^	0.82	1.87	2.646 (2)	158
N1—H1A···O2^ii^	0.86	2.35	3.167 (2)	160
C11—H11···O4	0.98	2.28	2.739 (2)	107

Symmetry codes: (i) *x*+1, *y*, *z*; (ii) −*x*+2, −*y*+1, −*z*+1.
